# Supplementary material for: An avalanche-and-surge robust ultrawide-bandgap heterojunction for power electronics
Source: Nat Commun. 2023 Jul 25;14:4459. doi: 10.1038/s41467-023-40194-0 (PMC10368629; doi:10.1038/s41467-023-40194-0)
Supplement: Supplementary file 1 — Supplementary Information [file 41467_2023_40194_MOESM1_ESM.pdf]

Supplementary Information

An avalanche-and-surge robust ultrawide-bandgap heterojunction for power electronics

Feng Zhou[1#], Hehe Gong[1#], Ming Xiao[2#], Yunwei Ma[2], Zhengpeng Wang[1], Xinxin Yu[1], Li Li[3,4], Lan Fu[4], Hark Hoe Tan[3,4], Yi Yang1, Fang-Fang Ren[1], Shulin Gu[1], Youdou Zheng[1], Hai Lu[1†], Rong Zhang[1†], Yuhao Zhang[2†], and Jiandong Ye[1†]

[1] *School of Electronic Science and Engineering, Nanjing University, 210008, Nanjing, China*

[2] *Center for Power Electronics Systems, Virginia Polytechnic Institute and State University, Blacksburg, 24060, VA, USA*

[3] *Australian National Fabrication Facility ACT Node, The Australian National University, Canberra, ACT 2601, Australia*

[4] *ARC Centre of Excellence for Transformative Meta-Optical Systems, Department of Electronic Materials Engineering, Research School of Physics, The Australian National University, Canberra, ACT 2600, Australia*

[#]Feng Zhou, Hehe Gong and Ming Xiao contributed equally to this work

† *Corresponding Author E-mails:*

hailu@nju.edu.cn, rzhang@nju.edu.cn, yhzhang@vt.edu and yejd@nju.edu.cn

Supplementary Section S1 - Device Fabrication Process

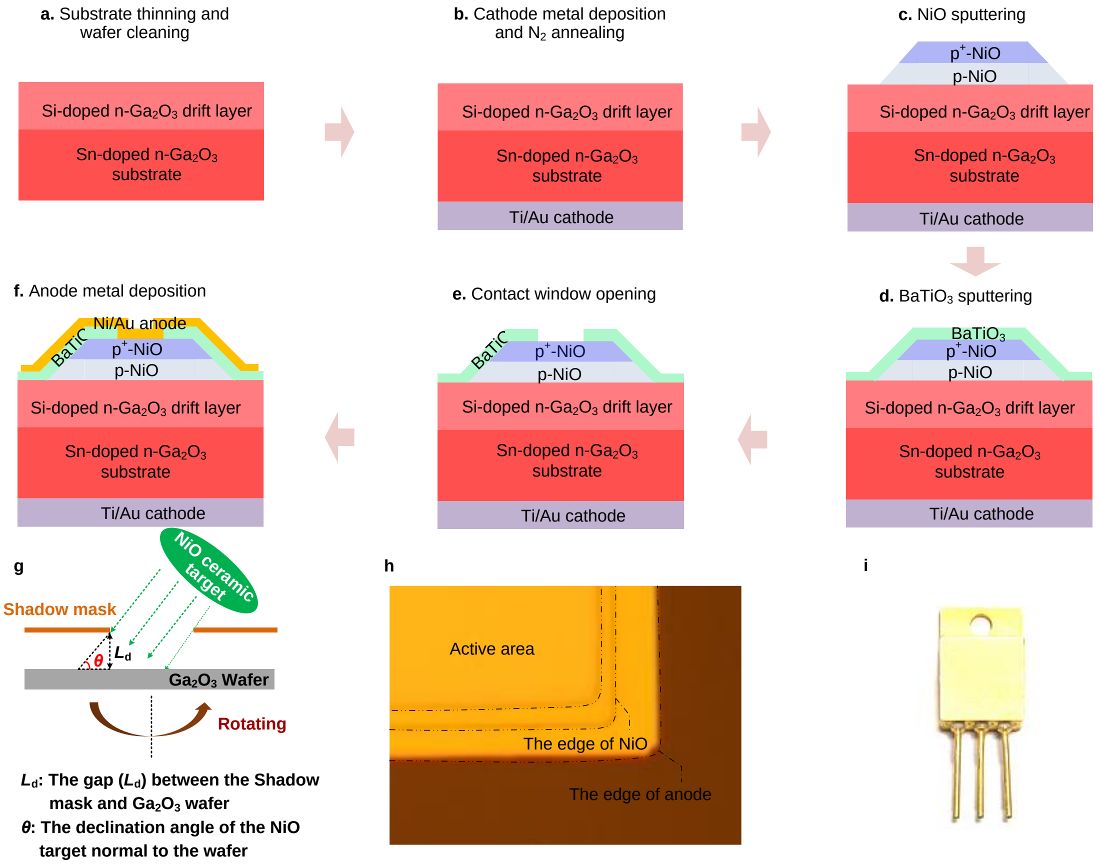

Supplementary Figure S1. Process flow of NiO/Ga_2O_3 HJD fabrication. The β-Ga_2O_3 (001) epi-wafer used in this work was from the commercial vendor in Japan, the Novel Crystal Technology (NCT). The provided datasheet shows that the substrate is the conductive Sn-doped (001) β-Ga_2O_3 grown by edge-defined film-fed growth (EFG) technique with an electron concentration of about 6×10^{18} cm^{-3} and a resistivity of 5×10^{-2} Ω·cm. A 10-μm-thick lightly-doped homoepitaxial layer was grown by halide vapor phase epitaxy (HVPE) technique. By performing capacitance-voltage (*C*-*V*) experiments, the net doping concentration of the epilayer is averaged to be 1.7×10^{16} cm^{-3}. The mobility value of epilayer was reported to be 145 cm^2/V s with a carrier concentration of ~10^{16} cm^{-3}, as measured by Hall test at room temperature [1]. The main fabrication steps of the NiO/Ga_2O_3 HJDs include (a) substrate thinning and wafer cleaning, (b) cathode metal deposition and N_2 annealing, (c) double-layered NiO deposition by RF sputtering, (d) $BaTiO_3$ deposition by RF sputtering, (e) anode contact window opening, (f) anode metal deposition, (g) Schematic showing the formation of the beveled angle in NiO thin films, which is adjustable by tuning the gap (L_d) between the shadow mask and Ga_2O_3 wafer as well as the declination angle (θ) of the NiO target normal to the Ga_2O_3 wafer, and (h) Optical microscope image of the anode region. The deposition of NiO double-layer and $BaTiO_3$ were both performed by using RF magnetron sputtering technology under the identical ambient gas (Ar/O_2) [2]. During the NiO sputtering process, the sample was rotated at a speed of 4 rpm to enhance the film uniformity. The growth pressure was 0.6 Pa in an Ar/O_2 mixed ambient, and the flux ratio of Ar/O_2 was tuned to modulate hole concentrations of NiO [3]. The fabricated large-area (3×3 mm^2) device was bonded on a 0.5-mm thick copper-molybdenum-copper (CMC) substrate with a high thermal conductivity of ~3$Wcm^{-1}K^{-1}$ [4], and the device cathode was connected to the lead frame by five 1-mm-diameter Au bonding wires to support the high surging current, which were ultimately encapsulated in the silicone-based potting compounds for TO-220 package [5], as shown in Fig. S1 (i).

Supplementary Section S2 - Capacitance-Voltage (*C-V*) Characterization

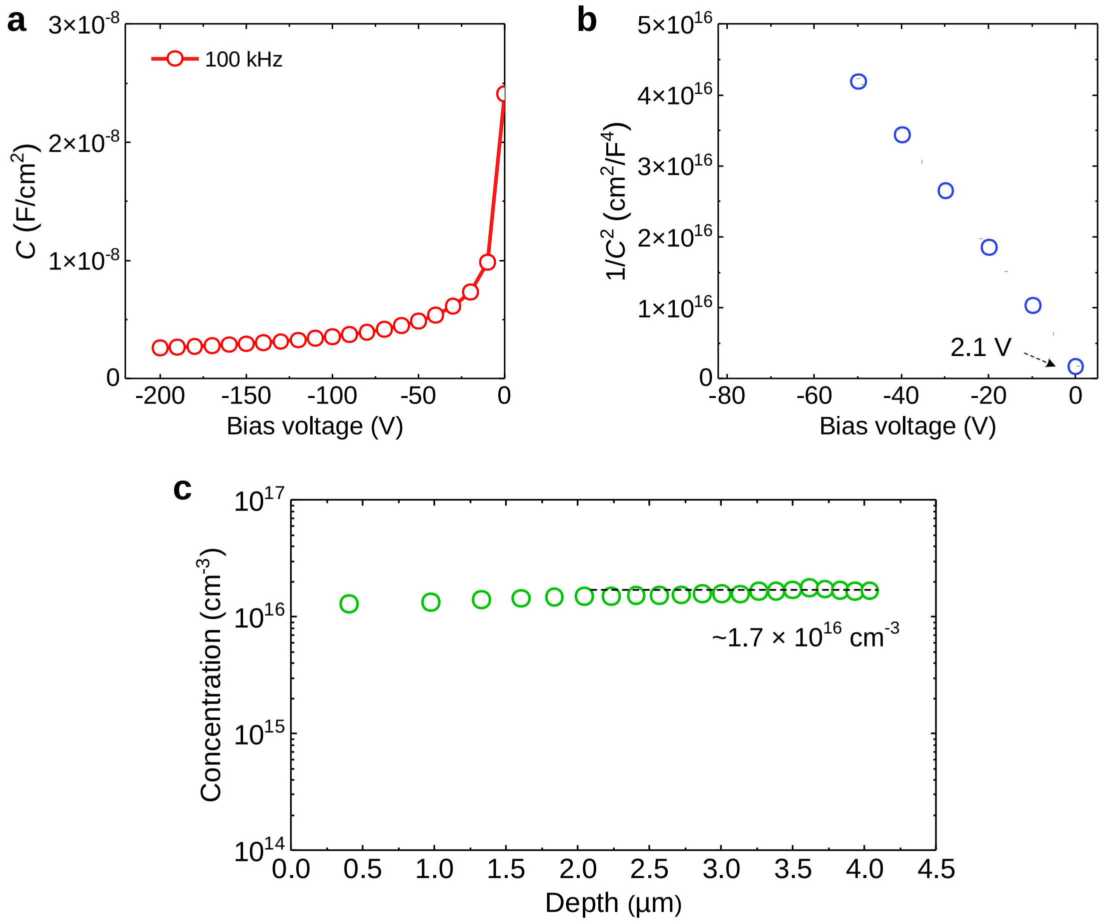

Supplementary Figure S2. Capacitance-Voltage (*C-V*) characterization. (a) *C-V* and (b) $1/C^2$-V characteristics of the HJD device measured at the frequency of 100 kHz at room temperature. (c) The extracted depth profile of net doping concentration (N_D - N_A) in the *n*-Ga_2O_3 drift layer from the *C-V* curve. The built-in potential (V_{bi}) is determined to be 2.1 V from the intercept of $1/C^2$-V plot on the *x*-axis [6]. The depth profile of net doping concentration (N_D - N_A) in the *n*-Ga_2O_3 drift layer is extracted from *C-V* curve, and the average net (N_D - N_A) is determined to be about 1.7×10^{16} cm^{-3}, which was used in the TCAD simulation.

Supplementary Section S3 - Experimental Circuit Testing Setups for Avalanche, Surge Current and Reverse Recovery Characterizations

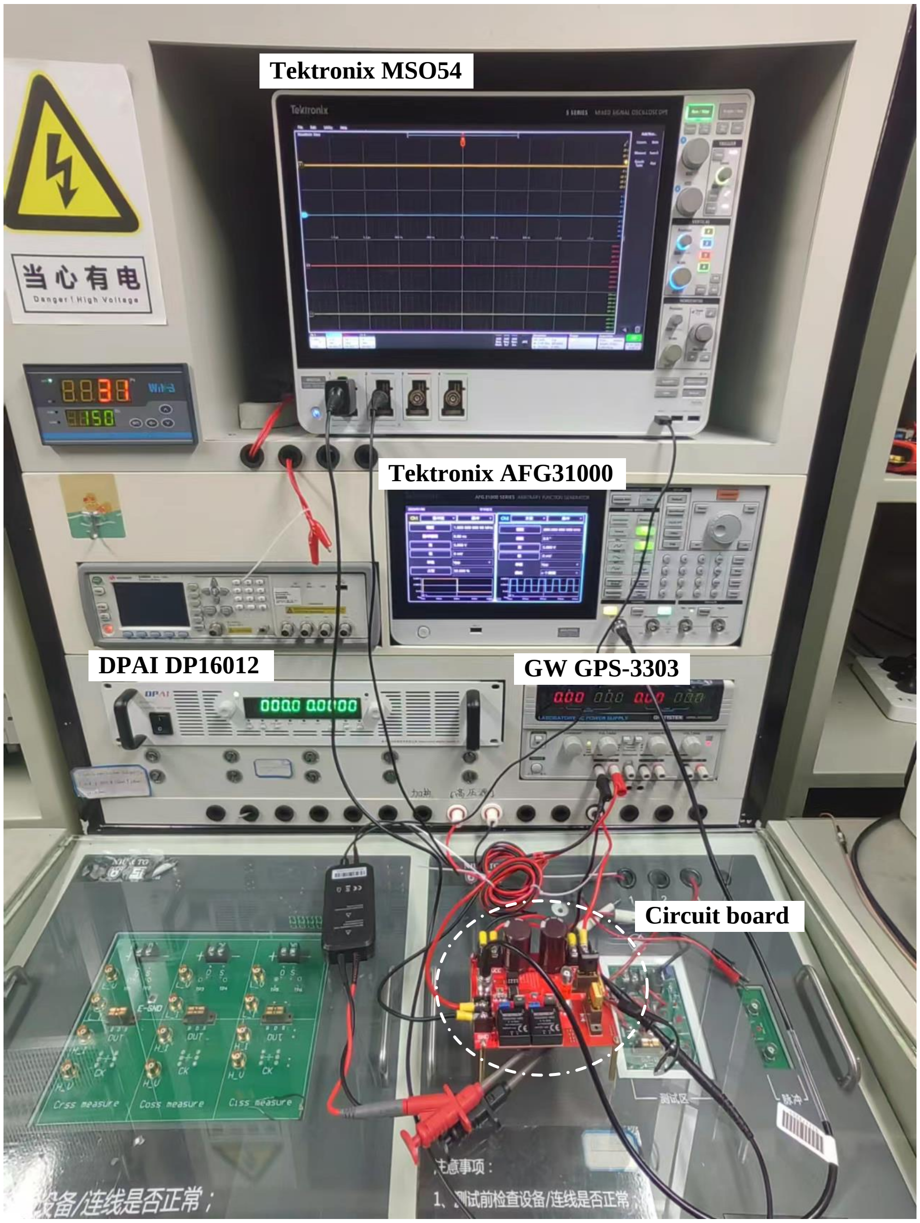

Supplementary Figure S3. Experimental circuit setups. Photographs of the experimental platform for avalanche, surge current and reverse recovery characterizations are shown, which consists of an MSO54 oscilloscope (Tektronix), an AFG31000 signal generator with built-in double pulse sequence (Tektronix), a DP16012 high voltage power supply (DPAI), a GPS-3303 DC power supply (GW INSTEK), and a printed circuit board for different tests. The high-voltage probe PCB5500B (5 kV, 500 MHz) from Zhiyong Electronics with a 100-fold voltage attenuation has been employed to record the voltage waveforms. Both the coaxial shunt (SSDN-414-10) and current probes (TCP0030A: 120 MHz, 0 ~ 30 A and TCP0150: 20 MHz, 0 ~ 150 A) have been used to measure the current waveforms. To maintain measurement accuracy, the current probe was degaussed before measurements.

Avalanche characterizations

The device under test (DUT) was reversely biased and connected in parallel with a high-BV_{AVA} SiC MOSFET [7]. In the test, the load inductor (L_{UIS}) was first charged by the power supply (V_{CC}) with the ON-state SiC MOSFET. Once the SiC MOSFET was turned OFF, the energy stored in L_{UIS} was forced to go through the DUT, driving it into the breakdown state, whereby the voltage and current waveforms were captured by the oscilloscope. By controlling a programmable pulse generator to adjust the ON/OFF state of the SiC MOSFET, the DUT could be driven into breakdown once-only or repeatedly. The SiC MOSFET is 1.7-kV rated (Wolfspeed, C2M0045170D); it did not avalanche due to its higher avalanche breakdown voltage. L_{UIS} with values from 50 μH to 30 mH were varied to modulate I_{AVA} and avalanche energy. The DC bus voltage (V_{DC}) was set to be 50~300 V.

Surge current characterizations

The DUT was forwardly biased and connected in series with a 72-A rated SiC MOSFET (Wolfspeed, C2M0045170D) [8]. In the test, a 10-ms-wide half-sinusoidal surge current was generated by the resonant circuit module and passed through the DUT. The resonant circuit module consists of a load inductor (L_{surge}) and a capacitor (C_{surge}). The corresponding surge current and voltage waveforms were recorded by the oscilloscope. The peak current (I_{peak}) can be tuned by adjusting the V_{CC}, C_{surge} and L_{surge}, in terms of the relationship of $I_{peak} = V_{CC} (C_{surge} / L_{surge})^{1/2}$ [9].

Reverse recovery characterizations

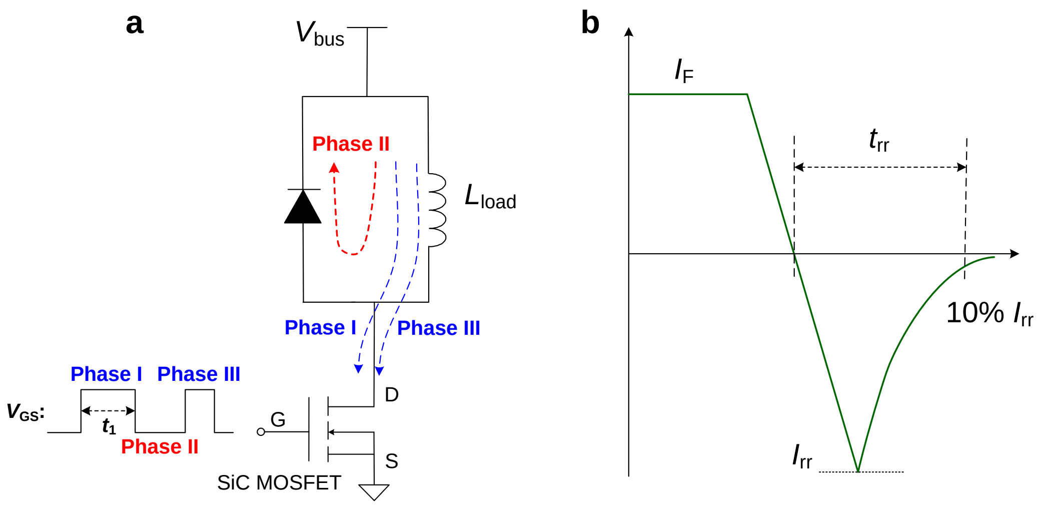

Supplementary Figure S4. Schematic of reverse recovery tests. (a) The schematic diagram of the double-pulse-test (DPT) operation principle. (b) Extraction of the reverse recovery time. A DPT circuit with inductive loads is widely used to measure the reverse recovery characteristics of diodes [10]. During the test, a SiC MOSFET [7] is first turned on to build up the linear inductor current (i.e., the desired forward current, I_F) in phase I, and I_F can be changed by adjusting the L_{load}, V_{bus} and t_1, according to the relationship of $I_F = (V_{bus} \times t_1) / L_{load}$, where t_1 is the first pulse width of the double pulse signal, L_{load} is the load inductor. When the SiC MOSFET is turned off (phase II), the DUT is under forward bias [11]. As the SiC MOSFET is turned on again (phase III), the diode switches to a reverse blocking condition with a short period of time (i.e., reverse recovery time, t_{rr}) usually present to remove the stored charge (i.e., reverse recovery charge). The t_{rr} is defined as the time when the reverse current recovers to 10% of its peak value (I_{rr}) (Fig. S4 (b)) [12]. Note that the second pulse width (phase III) is shorter than the first pulse width (phase I) to avoid overheating of the device.

Supplementary Section S4 – Physics-based Simulation of NiO/Ga_2O_3 Heterojunction

The physics-based Technology Computer Aided Design (TCAD) simulation is performed using Silvaco [13]. Table S1 lists the basic material parameters used in the simulation for the NiO/Ga_2O_3 heterojunction diode.

Table S1 Basic material parameters used in the simulations

Material	Key parameters
Ga_2O_3	Permittivity (ε_{GaO}):12.4 [plane (001)] [14]<br>Band-gap: 4.8 eV [15]<br>Affinity: 4.1 eV [16]
NiO	Permittivity (ε_{NiO}): 11.9 [17]<br>Band-gap: 3.8 eV [18]<br>Affinity: 2.0 eV [19]
$BaTiO_3$	Permittivity (ε_{BaTiO3}): 260 [20]<br>Band-gap: 3.4 eV [21]

Impact Ionization Coefficient Calculation

The Chynoweth model is used for the impact ionization (I. I.) coefficient of the electron (α_n) and hole (α_p), which formulates the I. I. coefficient (α) as Eqn. (S1),

$$\alpha = A \times e \frac{-B}{E} \quad \text{(S1)}$$

where E is electric field, A_n, B_n, A_p and B_p are constants related to the material and the subscript "n" and "p" represent electron and hole, respectively.

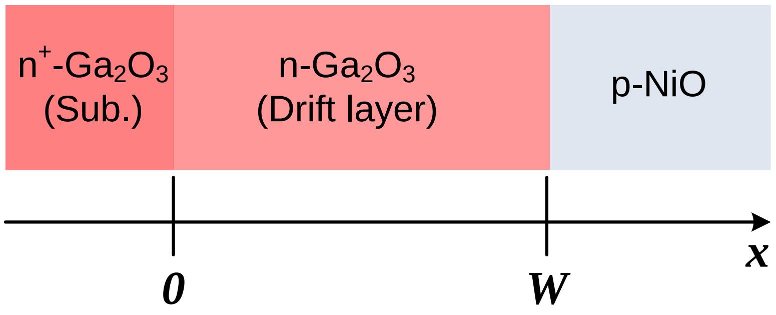

Supplementary Figure S5. Schematic of p-NiO/n-Ga_2O_3/n^+-Ga_2O_3 structure.

Figure S5 shows the schematic of the simplified 1-D model for the p-NiO/n-Ga_2O_3/n^+-Ga_2O_3 HJD. The relation between α and multiplication coefficient (M) is given by Eqn. (S2).

$$M_{(x)}=\frac{\exp\left[\int_0^x\left(\alpha_\mathrm{n}-\alpha_\mathrm{p}\right)\mathrm{d}x\right]}{1-\int_0^W\alpha_\mathrm{p}\exp\left[\int_0^x\left(\alpha_\mathrm{n}-\alpha_\mathrm{p}\right)\mathrm{d}x'\right]\mathrm{d}x} \tag{S2}$$

where W is the width of depletion region. Considering that the acceptor concentration (N_A > 10^{18} cm^{-3}) in p-NiO is much higher than the donor concentration (N_D ~ 1.7 × 10^{16} cm^{-3}) in the n^--Ga_2O_3 drift layer, the avalanche breakdown occurs in the punch-through condition, where n^--Ga_2O_3 is fully depleted. The punch-through voltage (V_punch) is calculated as

$$V_\mathrm{punch}=\frac{1}{2}\times\frac{qN_\mathrm{D}}{\varepsilon_\mathrm{GaO}}W\times W\approx 1240\ (V) \tag{S3}$$

where ε_GaO is the permittivity of Ga_2O_3. Note that the experimental avalanche voltage is ~1600 V, being higher than V_punch. With $V \geq V_\mathrm{punch}$, the E-field $E_{(x,V)}$ can be written as:

$$E_{(x,V)}=\frac{qN_\mathrm{D}}{\varepsilon_\mathrm{GaO}}x+\frac{V-V_\mathrm{punch}}{W},\ (V\geq V_\mathrm{punch}) \tag{S4}$$

Considering $x = W$ and $V \geq V_\mathrm{punch}$ and substituting Eqn. (S1) and (S4) into Eqn. (S2), the multiplication coefficient can be calculated as:

$$M_{(V)}=\frac{\exp\left\{\int_0^W\left[A_\mathrm{n}\exp\left(-\frac{B_\mathrm{n}}{\frac{qN_\mathrm{D}x}{\varepsilon_\mathrm{GaO}}+\frac{V-V_\mathrm{punch}}{W}}\right)-A_\mathrm{p}\exp\left(-\frac{B_\mathrm{p}}{\frac{qN_\mathrm{D}x}{\varepsilon_\mathrm{GaO}}+\frac{V-V_\mathrm{punch}}{W}}\right)\right]dx\right\}}{1-\int_0^W\left\{A_\mathrm{p}\exp\left(-\frac{B_\mathrm{p}}{\frac{qN_\mathrm{D}x}{\varepsilon_\mathrm{GaO}}+\frac{V-V_\mathrm{punch}}{W}}\right)\int_0^{x'}\left[A_\mathrm{n}\exp\left(-\frac{B_\mathrm{n}}{\frac{qN_Dx}{\varepsilon_\mathrm{GaO}}+\frac{V-V_\mathrm{punch}}{W}}\right)-A_\mathrm{p}\exp\left(-\frac{B_\mathrm{p}}{\frac{qN_\mathrm{D}x}{\varepsilon_\mathrm{GaO}}+\frac{V-V_\mathrm{punch}}{W}}\right)\right]dx'\right\}dx} \tag{S5}$$

According to Eqn. (S5), M is a function of V and the fitting parameters in Chynoweth I. I. model for electrons and holes (A_n, B_n, A_p, and B_p).

The multiplication coefficient M can be extracted from the experimental avalanche *I-V* characteristics (Fig. 2(a)), as defined by the ratio between the avalanche current (I_total) and background current ($I_\mathrm{background}$) before avalanche [22]. As shown in Figure S6, a polynomial fit between 0 and 1400 V was extended to the avalanche voltage range to obtain $I_\mathrm{background}$. The avalanche multiplication effect causes the background electrons/holes to multiply, making the I_total. Using this method, *M~V* characteristics can be obtained from experimental *I-V* characteristics.

The values of A_n and B_n are theoretically predicted in Ref. [23], which are used as the initial numbers in the fitting. A_p and B_p can be obtained by the method of least squares to fit the *M-V* characteristics derived from the experimental *I-V* characteristics. As no A_p and B_p have been reported, we choose the initial values of A_p and B_p to be similar to A_n and B_n. Figure S7 shows the calculated and experimental results of M, revealing a good agreement. Note that the slight deviation of the model at high avalanche currents is due to non-idealities related to the material and device structure (e.g., series resistance). The extracted key parameters for the Chynoweth model are summarized in Table S2.

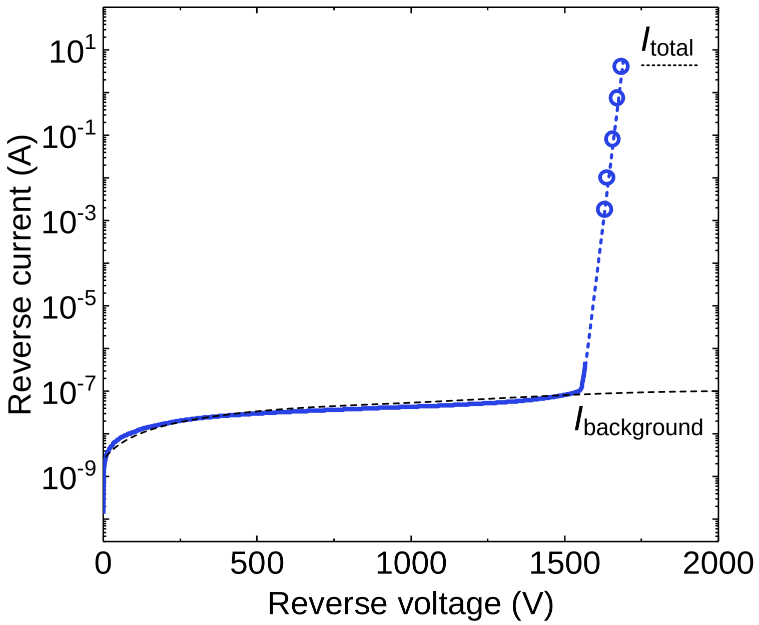

Supplementary Figure S6. The extraction of *M* from experimental *I-V* characteristics by dividing the total current over the background current. Background current is fitted between 0 V to 1400 V before avalanche occurs.

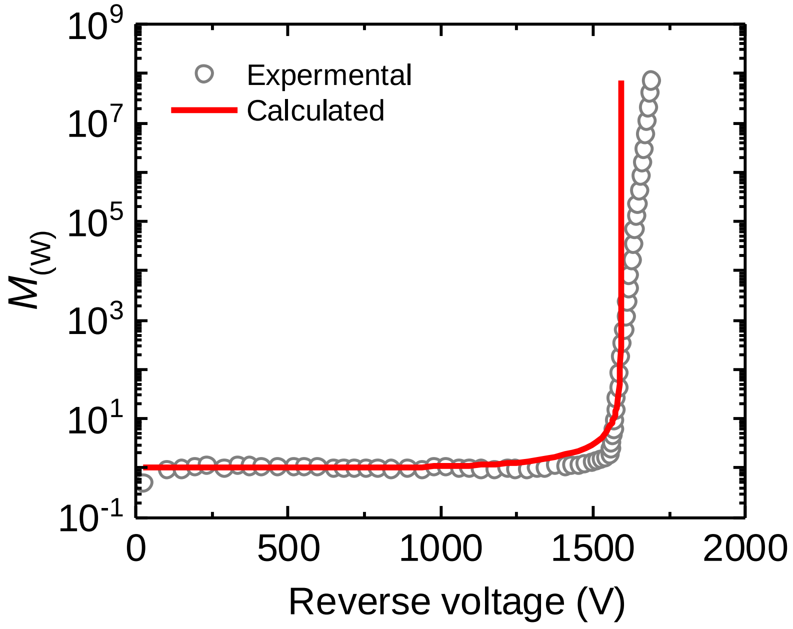

Supplementary Figure S7. Experimental and calculated multiplication coefficient characteristics; the calculation is based on the Chynoweth model for I. I. coefficients with the extracted A_n, B_n, A_p, and B_p parameters in Table S2.

Table S2 Key model and parameters in the avalanche and surge-current simulations

Parameter	Model	Key parameters
Ga_2O_3 Impact Ionization Coefficient Models	Chynoweth Impact Ionization Model $\alpha_n = A_n \times e^{\frac{-B_n}{E}}, \alpha_p = A_p \times e^{\frac{-B_p}{E}}$	A_n=2.16×10^6 cm^{-1} B_n=1.77×10^7 V/cm A_p=5.75×10^6 cm^{-1} B_p=1.77×10^7 V/cm
Ga_2O_3 & NiO minority carrier mobility model	Electric Field-Dependent Mobility model $\mu_n = \mu_{n0}\left[\frac{1}{1+(\frac{\mu_{n0}E}{\mu_{satn}})}\right]^{\frac{1}{2}}, \mu_p = \mu_{p0}\left[\frac{1}{1+(\frac{\mu_{p0}E}{\mu_{satp}})}\right]^{\frac{1}{2}}$	$\mu_{n0,GaO}$ =145 cm^2/V s (drift region) $\mu_{p0,GaO}$ =1 cm^2/V s (drift region) [24] $\mu_{n,GaO}$ =25 cm^2/V s (substrate) $\mu_{p,GaO}$ =1 cm^2/V s (substrate) $\mu_{n0,NiO}$ =0.6 cm^2/V s [25] $\mu_{p0,NiO}$ =0.87 cm^2/V s (from Hall measurement)

Avalanche simulation and mobility model

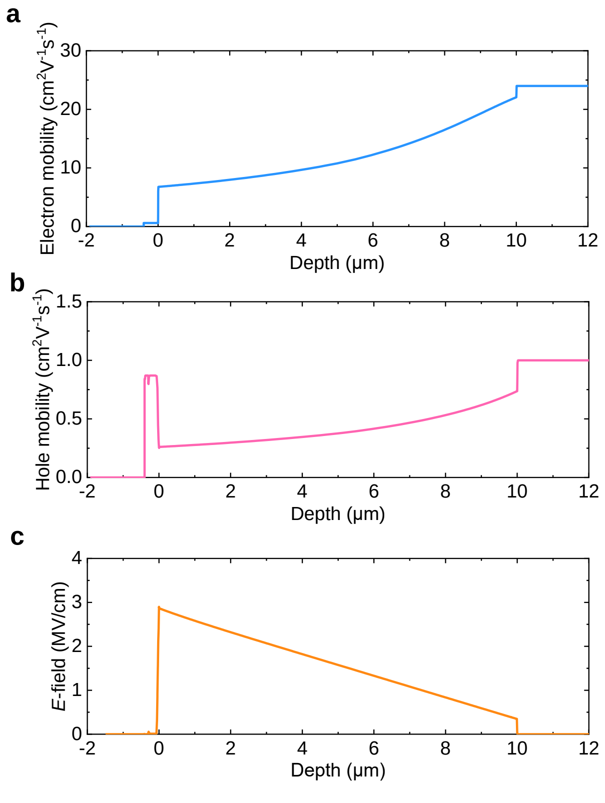

Supplementary Figure S8. Simulated profile of carrier mobilities and *E*-field under the avalanche condition.

Figure S8 shows the simulated profile of (a) electron and (b) hole mobilities and (c) *E*-field as a function of vertical depth in the HJD under an I_{AVA} of 30 A using the models and parameter in Table S2. In the drift region, carrier mobilities increase from the heterojunction towards the substrate due to the reduced *E*-field. In the Ga_2O_3 substrate, the *E*-field is nearly zero, leading to constant carrier mobilities.

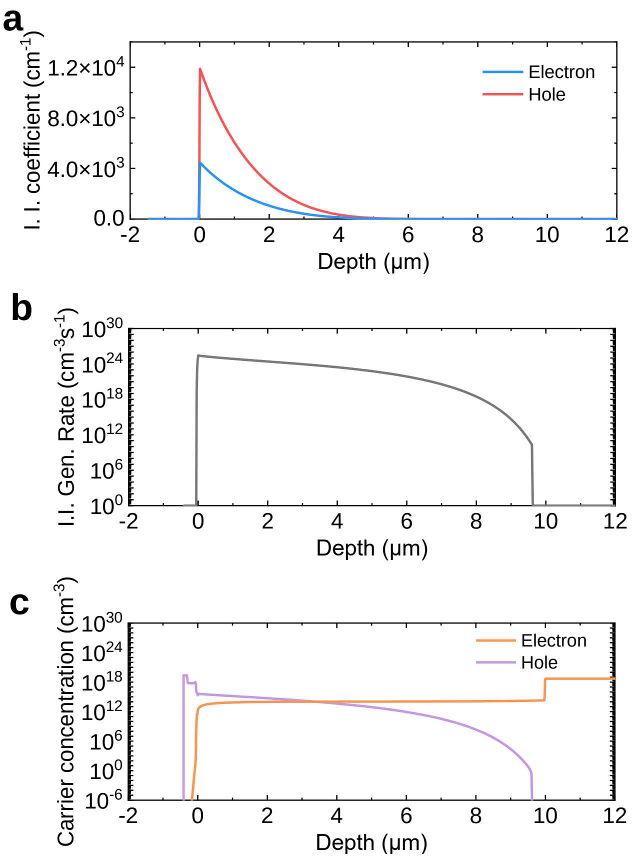

Supplementary Figure S9. Simulated profiles of the I. I. coefficients, I. I. generation rates and carrier concentrations under the avalanche condition.

Figure S9 shows the simulated profile of (a) I. I. coefficient, (b) I. I. generation rate, and (c) carrier concentrations as a function of vertical depth in the HJD under an I_{AVA} of 30 A using the models and parameter in Table S2. The I. I. coefficients and generation rates show strong dependence on E-field, both peaking at the heterojunction. This is consistent with the analytical calculation presented above. In addition, Fig. S9(a) suggests that the I. I. coefficient of hole is higher than that of electron in Ga_2O_3 drift region, which is consistent with the cases in SiC and GaN materials [26]. Note that the I. I. coefficient is influenced by multiple material properties including the material bandgap, electric field, optical phonon energy, mean free path, electron mobility and electric field, as suggested by the widely-used Thornber model [27]. Fig. S9(b) shows that the I. I. generation rate in Ga_2O_3 is much higher than in NiO, confirming the initiation of I. I. in Ga_2O_3.

Surge simulations

The basic parameters and key minority carrier mobility used in the simulation are shown in Table S1 and S2, respectively. Other physical models and parameters related to minority carrier transport include the carrier recombination, minority carrier mobility, and minority carrier lifetime models. The Shockley-Read-Hall (SRH) recombination model is employed for the recombination at the heterojunction interface. All minority carrier lifetime parameters are extracted at room temperature and low electric field. The hole lifetime ($\tau_{h,GaO}$) of 6.2 ns in n-Ga_2O_3 and the electron lifetime in p-NiO ($\tau_{e,NiO}$) of 124.0 ns in p-NiO, respectively, were extracted from microscopic EBIC characterizations. These values are employed in the simulation.

Supplementary Section S5 – *I-V* characteristics the HJD before and after the repetitive avalanche tests

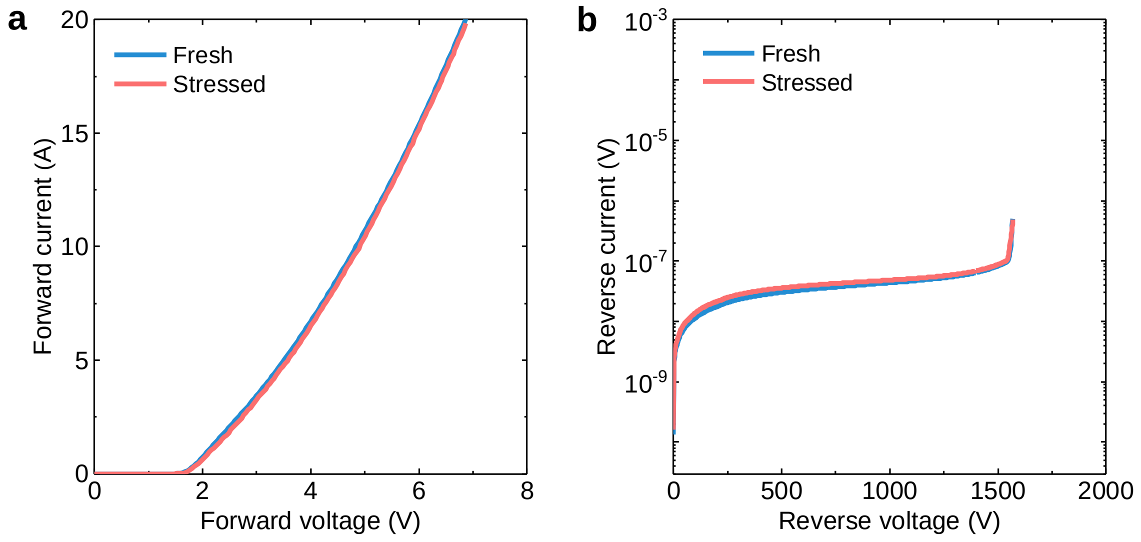

Supplementary Figure S10. (a) Forward and (b) reverse current-voltage (*I-V*) characteristics of the HJD before and after the 1 million times avalanche tests with a BV_{AVA} of 1740 V and a I_{AVA} of 30 A. The *I-V* curves of the device before and after this cycle testing show minimal parametric shifts.

Supplementary Section S6 - Surge Characteristics of the Reference Ga_2O_3 Schottky Barrier Diode

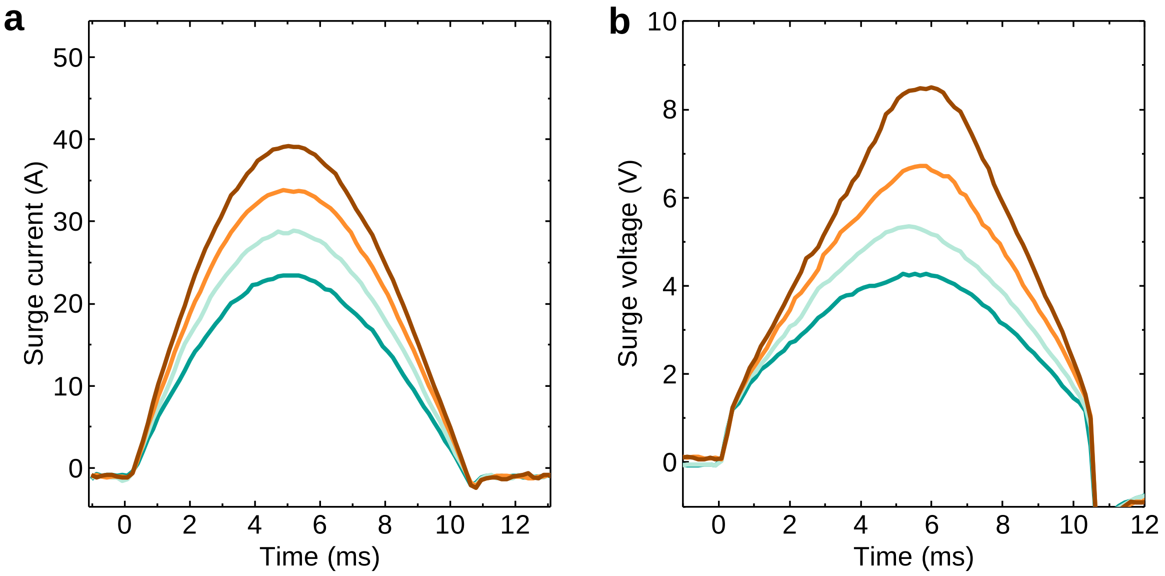

Supplementary Figure S11 Surge current characteristics of the Ga_2O_3 Schottky barrier diode (SBD). (a) Surge current and (b) surge voltage curves of the SBD. The measurement setup of the surge current is shown in Supplementary Fig. S4.

Supplementary Section S7 - Log-Scale Forward *I-V-T* Characteristics of the NiO/Ga_2O_3 HJD

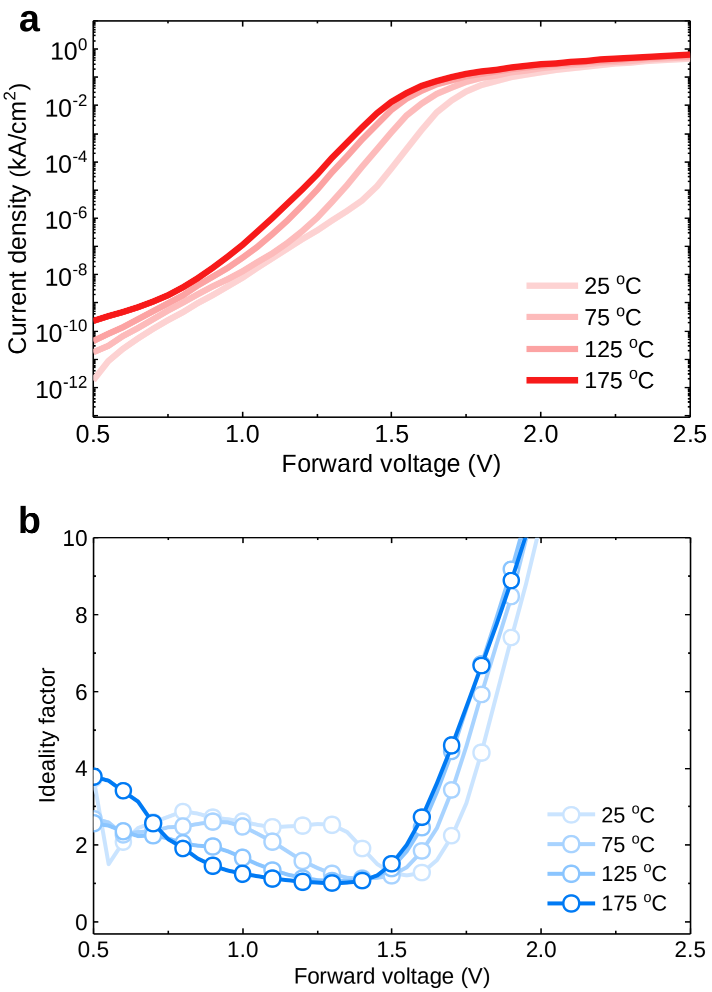

Supplementary Figure S12. Log-scale forward *I-V-T* characteristics. (a) *T*-dependent forward *I-V* characteristics and (b) extracted ideality factors of the Ga_2O_3 HJD. These results were measured in the DC mode of the B1505 power device analyzer. In the subthreshold region, the ideality factor of the device is close to 2, suggesting that the interfacial SRH recombination mechanism is dominant at low forward bias. The increased subthreshold current at higher temperature is a result of high temperature-induced lowering of the barrier height.

Supplementary Section S8 - Current-voltage Characteristics of the p-NiO layer

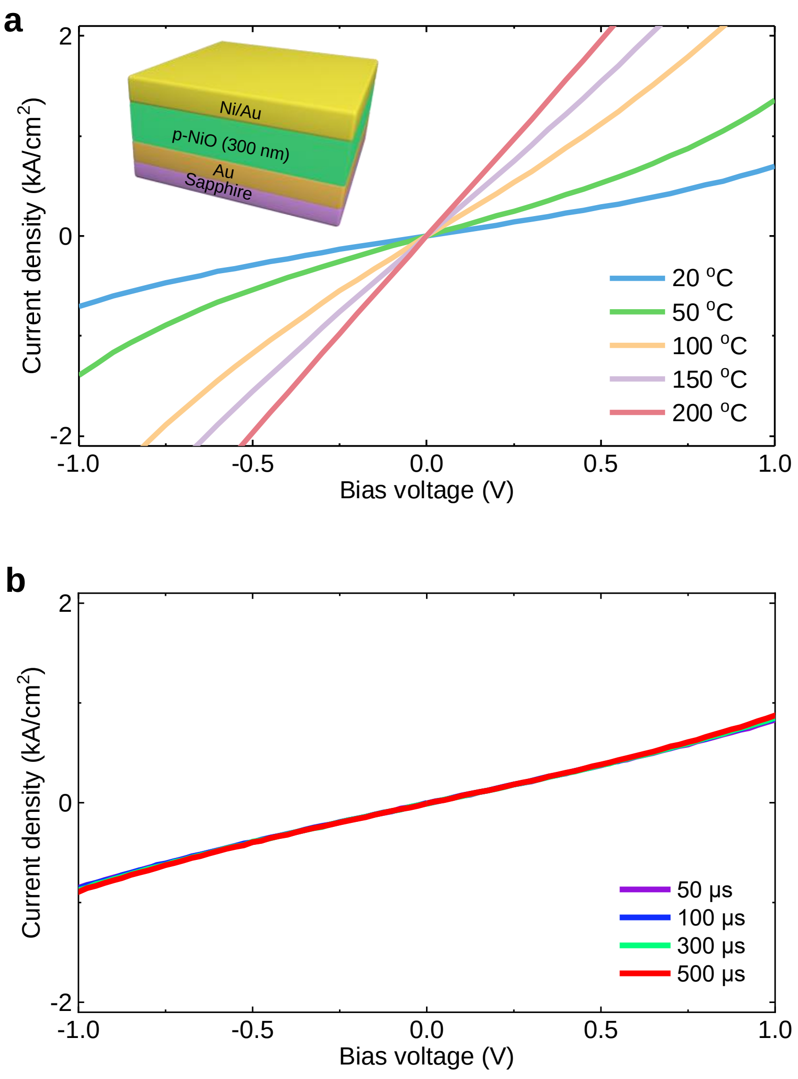

Supplementary Figure S13. Current-voltage characteristics of the metal-NiO-metal (MSM) structure at different (a) temperatures and (b) pulse widths. To evaluate the contribution of p-NiO on the conductivity modulation of the entire NiO/Ga_2O_3 p-n heterojunction, *I-V* characterizations for the NiO films under different temperatures (T_s) and pulse widths (*PW*s) have been performed and shown in Fig. S12 (a) and (b). Linear *I-V* features with large current densities are observed, indicating the nature of Ohmic contact to NiO by Ni/Au metal stacks. Note that the current density is almost unchanged under different *PW*s, which is evidently different from the *PW*-dependent *I-V* characteristics of HJD. This implies that the enhanced current capability of HJD is not mainly contributed by the intrinsic characteristic change of NiO but tightly related to conductivity modulation. Note that, at high temperatures, increased acceptor ionization in p-NiO and decreased contact resistance could both contribute to the improved current conduction.

Supplementary Section S9 - Benchmarking $R_{on,sp}$ versus *BV* of the ampere-class Ga_2O_3 HJD against small-area and large-area Ga_2O_3 SBDs, HJDs and junction barrier Schottky diode (JBSs).

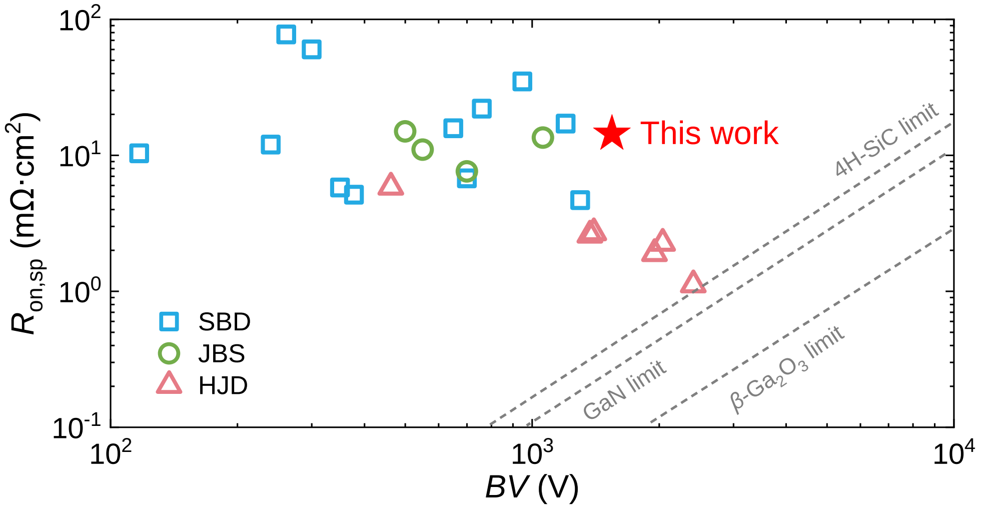

Supplementary Figure S14. $R_{on,sp}$ versus *BV* benchmark for Ga_2O_3 SBDs, HJDs and JBS diodes [4-6], [12], [29-38]. Note that ampere-level large-area power devices are required for industrial applications, so their performance usually represents the application prospects of a power device technology. For the emerging Ga_2O_3 power technology, there is still a large gap between the performance of large-area devices and small-area devices, possibly due to the non-uniformity in fabrication process and wafer material properties. In addition, the breakdown voltage measured under the switching circuit tests instead of static *I-V* tests represents the true overvoltage margin of devices in practical power electronics applications. Note that, in this benchmark, the *BV* of devices in this work is the only one that has been validated in the circuit tests. The robust breakdown capability achieved in this work is attributed to the effective edge termination that incorporates the small-angle beveled junction termination extension (JTE) and high-k field plate.

References

[1] Goto, K. *et al.* Halide vapor phase epitaxy of Si doped β-Ga_2O_3 and its electrical properties. *Thin Solid Films* **666**, 182-184 (2018).

[2] Gong, H. H. *et al.* A 1.86-kV double-layered NiO/β-Ga_2O_3 vertical p–n heterojunction diode. *Appl. Phys. Lett.* **117**, 022104 (2020).

[3] Luo, H. *et al.* Fabrication and Characterization of High-Voltage NiO/β-Ga_2O_3 Heterojunction Power Diodes. *IEEE Trans. Electron Devices* **68**, 3991-3996 (2021).

[4] Gong, H. *et al.* 70-µm-Body Ga_2O_3 Schottky Barrier Diode with 1.48 K/W Thermal Resistance, 59 A Surge Current and 98.9% Conversion Efficiency. *IEEE Electron Device Lett.* **43**, 773-776 (2022).

[5] Zhou, F. *et al.* 1.95-kV Beveled-mesa NiO/Ga_2O_3 Heterojunction Diode with 98.5% Conversion Efficiency and Over Million-Times Overvoltage Ruggedness. *IEEE Trans. Power Electron.* **37**, 1223-1227 (2021).

[6] Zhou, F. *et al.* Over 1.8 GW/cm^2 beveled-mesa NiO/β-Ga_2O_3 heterojunction diode with 800 V/10 A nanosecond switching capability. *Appl. Phys. Lett.* **119**, 262103 (2021).

[7] Liu, J. *et al.* Trap-mediated avalanche in large-area 1.2 kV vertical GaN p-n diodes. *IEEE Electron Device Lett.* **41**, 1328-1331 (2020).

[8] Liu, J. *et al.* Surge Current and Avalanche Ruggedness of 1.2 kV Vertical GaN p-n Diodes. *IEEE Trans. Power Electron.* **36**, 10959-10964 (2021).

[9] Liu, Y., Yang, S., Han, S. & Sheng, K. Investigation of Surge Current Capability of GaN E-HEMTs in The Third Quadrant: The Impact of P-GaN Contact. *IEEE J. Emerg. Sel. Topics Power Electron.* **7**, 1465-1474 (2019).

[10] Chen, K. J. *et al.* GaN-on-Si power technology: devices and applications. *IEEE Electron Device Lett.* **64**, 779-795 (2017).

[11] Zhou, F. *et al.* Demonstration of Avalanche and Surge-Current Robustness in GaN Junction Barrier Schottky Diode with 600 V/10 A Switching Capability. *IEEE Trans. Power Electron.* **36**, 12163-12167 (2021).

[12] Gong, H. *et al.* 1.37 kV/12 A NiO/β-Ga_2O_3 heterojunction diode with nanosecond reverse recovery and rugged surge-current capability. *IEEE Trans. Power Electron.* **36**, 12213-12217 (2021).

[13] ATLAS User's Manual: Device Simulation Software Datasheet; https://www.eng.buffalo.edu/~wie/silvaco/atlas_user_manual.pdf.

[14] Fiedler, A., Schewski, R., Galazka, Z. & Irmscher, K. Static Dielectric Constant of β-Ga_2O_3 Perpendicular to the Principal Planes (100), (010), and (001). *ECS J. Solid State Sci. Technol.* **8**, Q3083-3085 (2019).

[15] Pearton, S. J. *et al.* A review of Ga_2O_3 materials, processing, and devices. *Appl. Phys. Rev.* **5**, 011301 (2018).

[16] Larsson, F. *et al.* Atomic layer deposition of amorphous tin-gallium oxide films. *J. Vac. Sci. Technol., A* **37**, 030906 (2019).

[17] Spencer, J. A. *et al.* A review of band structure and material properties of transparent conducting and semiconducting oxides: Ga_2O_3, Al_2O_3, In_2O_3, ZnO, SnO_2, CdO, NiO, CuO, and Sc_2O_3. *Appl. Phys. Rev.* **9**, 011315 (2022).

[18] Li, J.-S. *et al.* Demonstration of 4.7 kV breakdown voltage in NiO/β-Ga_2O_3 vertical rectifiers. *Appl. Phys. Lett.* **121**, 042105 (2022).

[19] Wu, H. & Wang, L.-S. A study of nickel monoxide (NiO), nickel dioxide (ONiO), and Ni(O_2) complex by anion photoelectron spectroscopy. *J. Chem. Phys.* **107**, 16-21 (1997).

[20] Rahman, M. W., Joishi, C., Kalarickal, N. K., Lee, H. & Rajan, S. Demonstration of $BaTiO_3$ Integrated kV-class AlGaN/GaN Schottky Barrier Diodes with Record Average Breakdown Electric Field. In *2022 IEEE 34th International Symposium on Power Semiconductor Devices and ICs.* 341-344 (IEEE, 2022); doi: 10.1109/ispsd49238.2022.9813622.

[21] Reddy, Y. K. V., Mergel, D., Reuter, S., Buck, V. & Sulkowski, M. Structural and optical properties of $BaTiO_3$ thin films prepared by radio-frequency magnetron sputtering at various substrate temperatures. *J. Phys. D: Appl. Phys.* **39**, 1161-1168 (2006).
[22] Konstantinov, A. O., Wahab, Q., Nordell, N. & Lindefelt, U. Study of avalanche breakdown and impact ionization in 4H silicon carbide. *J. of Electron. Mater.* **27**, 335-341 (1998).
[23] Ghosh, K. & Singisetti, U. Impact ionization in β-Ga_2O_3. *J. Appl. Phys.* **124**, 085707 (2018).
[24] Poncé, S. & Giustino, F. Structural, electronic, elastic, power, and transport properties of $\beta-Ga_2O_3$ from first principles. *Phys. Rev. Res.* **2**, 033102 (2020).
[25] Handley, S. J. & Bradberry, G. W. Estimates of charge carrier mobility and lifetime in nickel oxide. *Phys. Lett. A* **40**, 277-278 (1972).
[26] Cooper, J. A. & Morisette, D. T. Performance limits of vertical unipolar power devices in GaN and 4H-SiC. *IEEE Electron Device Letters* **41**, 892-895 (2020).
[27] Nouketcha, F. L. *et al.* Investigation of wide-and ultrawide-bandgap semiconductors from impact-ionization coefficients. *IEEE Transactions on Electron Devices* **67**, 3999-4005 (2020).
[28] Ji, M. *et al.* Demonstration of large-size vertical Ga_2O_3 Schottky barrier diodes. *IEEE Trans. Power Electron.* **36**, 41-44 (2021).
[29] Lv, Y. *et al.* Demonstration of β-Ga_2O_3 Junction Barrier Schottky Diodes with a Baliga's Figure ofMerit of 0.85 GW/cm^2 or a 5A/700 V Handling Capabilities. *IEEE Trans. Power Electron.* **36**, 6179-6182 (2020).
[30] Otsuka, F. *et al.* Large-size (1.7 $\times$ 1.7 mm^2) β-Ga_2O_3 field-plated trench MOS-type Schottky barrier diodes with 1.2 kV breakdown voltage and 109 high on/off current ratio. *Appl. Phys. Exp.* **15**, 016501 (2021).
[31] Sharma, R. *et al.* Effect of probe geometry during measurement of >100 A Ga_2O_3 vertical rectifiers. *J. Vac. Sci. Technol., A* **39**, 013406 (2021).
[32] Wei, J. *et al.* Experimental Study on Electrical Characteristics of Large-Size Vertical β-Ga_2O_3 Junction Barrier Schottky Diodes. In *2022 IEEE 34th International Symposium on Power Semiconductor Devices and ICs.* 97-100 (IEEE, 2022); doi: 10.1109/ispsd49238.2022.9813623.
[33] Xiao, M. *et al.* Packaged Ga_2O_3 Schottky Rectifiers with Over 60 A Surge Current Capability. *IEEE Trans. Power Electron.* **36**, 8565-8569 (2021).
[34] Yang, J. *et al.* Reverse Breakdown in Large Area, Field-Plated, Vertical β-Ga_2O_3 Rectifiers. *ECS J. Solid State Sci. Technol.* **8**, Q3159-Q3164 (2019).
[35] Yang, J., Ren, F., Tadjer, M., Pearton, S. J. & Kuramata, A. Ga_2O_3 Schottky rectifiers with 1 ampere forward current, 650 V reverse breakdown and 26.5 MW.cm^{-2} figure-of-merit. *AIP Adv.* **8**, 055026 (2018).
[36] Yang, J. *et al.* Vertical geometry 33.2 A, 4.8 MW cm^2 Ga_2O_3 field-plated Schottky rectifier arrays. *Appl. Phys. Lett.* **114**, 232106 (2019).
[37] Zhang, Y., Udrea, F. & Wang, H. Multidimensional device architectures for efficient power electronics. *Nat. Electron.* **5**, 723-734 (2022).
[38] Wang, Y. g. *et al.* 2.41 kV Vertical p-NiO/n-Ga_2O_3 Heterojunction Diodes with a Record Baligas Figure-of-Merit of 5.18 GW/cm^2. *IEEE Trans. Power Electron.* **37**, 3743-3746 (2022).
