# Supplementary material for: An avalanche-and-surge robust ultrawide-bandgap heterojunction for power electronics
Source: Nat Commun. 2023 Jul 25;14:4459. doi: 10.1038/s41467-023-40194-0 (PMC10368629; doi:10.1038/s41467-023-40194-0)
Supplement: Supplementary file 3 — Description of Additional Supplementary Files [file 41467_2023_40194_MOESM3_ESM.pdf]

**Description of Additional Supplementary Files**

File Name: Supplementary Movie 1
Description: Device package and circuit testing.
